# Prestige and content biases together shape the cultural transmission of narratives

**DOI:** 10.1017/ehs.2021.37

**Published:** 2021-07-29

**Authors:** Richard E.W. Berl, Alarna N. Samarasinghe, Seán G. Roberts, Fiona M. Jordan, Michael C. Gavin

**Affiliations:** 1 Department of Human Dimensions of Natural Resources, Colorado State University, Fort Collins, CO 80523-1480, USA; 2 Department of Anthropology and Archaeology, University of Bristol, Bristol, United Kingdom; 3 School of English, Communication and Philosophy, Cardiff University, Cardiff, United Kingdom; 4 Max Planck Institute for the Science of Human History, Jena, Germany

**Keywords:** cultural transmission, prestige, transmission biases, cognition, sociolinguistics, storytelling

## Abstract

Cultural transmission biases such as prestige are thought to have been a primary driver in shaping the dynamics of human cultural evolution. However, few empirical studies have measured the importance of prestige relative to other effects, such as content biases present within the information being transmitted. Here, we report the findings of an experimental transmission study designed to compare the simultaneous effects of a model using a high- or low-prestige regional accent with the presence of narrative content containing social, survival, emotional, moral, rational, or counterintuitive information in the form of a creation story. Results from multimodel inference reveal that prestige is a significant factor in determining the salience and recall of information, but that several content biases, specifically social, survival, negative emotional, and biological counterintuitive information, are significantly more influential. Further, we find evidence that reliance on prestige cues may serve as a conditional learning strategy when no content cues are available. Our results demonstrate that content biases serve a vital and underappreciated role in cultural transmission and cultural evolution.

**Social media summary:** Storyteller and tale are both key to memorability, but some content is more important than the storyteller's prestige.

## Graphical abstract

**Figure:**
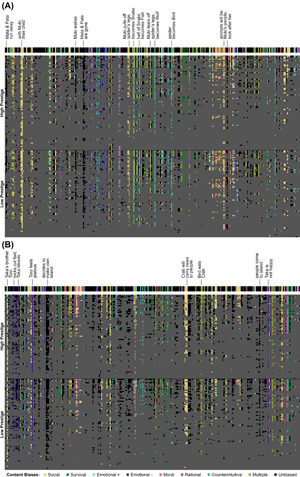

## Introduction

Storytelling is a powerful and universal tool that humans use to know and understand the world (Bruner, 1991, 2009), to preserve history and traditional knowledge (Vansina, 1985; Lejano et al., 2013), to educate (Cajete, 1994; Piquemal, 2003), to persuade (Chang, 2009; Delgadillo & Escalas, 2004), and to heal (Struthers et al., 2004; White et al., 1990). Stories encode complex cultural and ecological information, and have the capability to endure for thousands of years (da Silva & Tehrani, 2016; Nunn & Reid, 2016; Tehrani & d’Huy, 2017). In addition, skilled storytelling may increase an individual's reproductive fitness (Scalise Sugiyama, 1996; Smith et al., 2017) and social value, as well as promoting cooperation within groups (Smith et al., 2017).

Stories are an efficient and effective way to pass on valuable information from generation to generation (Boyd, 2009), but why do some stories endure while others burn out soon after they are told (Tehrani, 2013; da Silva & Tehrani, 2016)? Why do certain elements of stories persist over time while others grow, change, or disappear? The differential survival of stories and their constituent parts could depend on aspects that make them more attractive—such as their narrative qualities, themes, or content—or on the identities of the storytellers themselves—including their reputation within the community, their charisma, or their performative skill. A body of literature in the interdisciplinary field of cultural evolution suggests that the success or failure of a story and its components are determined by internal cognitive biases that favor either the content of the story or the context of its telling, including the speaker or “model” of the story (Barrett & Nyhof, 2001; Bebbington et al., 2017; Heath et al., 2001; Mesoudi et al., 2006; Stubbersfield et al., 2015; Stubbersfield & Tehrani, 2013). The biases that govern the transmission of stories from one person to another are thought to be the same that dictate the transmission of any kind of information, and these two specific types are referred to as “content” biases and “model-based” biases (Richerson & Boyd, 2005).

In this study, our goal is to evaluate the relative effects of content biases and model-based biases on cultural transmission and thereby test longstanding theories in the field of cultural evolution. Despite the critical role that transmission biases appear to play in driving cultural evolution, serious gaps exist in our understanding of the relative strengths of these biases (Acerbi & Mesoudi, 2015; McElreath et al., 2008; Kendal et al., 2018; Jiménez & Mesoudi, 2019). In particular, prior experimental studies have tended to focus on individual biases (Mesoudi & Whiten, 2008; Kendal et al., 2018), yet multiple biases are always present simultaneously (Heath et al., 2001; Stubbersfield et al., 2015; Atkisson et al., 2012; Morgan et al., 2012; Acerbi & Tehrani, 2018; Stubbersfield et al., 2019). Narrative stories are especially dense in information and contain a number of proposed content biases that have been shown to aid in the salience and retention of transmitted information (Mesoudi et al., 2006; Boyer & Ramble, 2001; Eriksson & Coultas, 2014; Nairne et al., 2007; Norenzayan & Atran, 2004).

Content biases influence transmission through properties of the information itself that make it more appealing and memorable (Boyd & Richerson, 1985). Preferences for certain types of information can vary between individuals and across cultures, but some have been seen to be remarkably consistent (Barrett & Broesch, 2012). Here, we conduct the first simultaneous test of the relative effects of the most frequently cited content biases from the cultural evolution literature. This includes content linked to the following six types of information: (*i*) *social*, either in the sense of everyday basic social interaction or of “gossip” about third parties (Mesoudi et al., 2006; Stubbersfield et al., 2015); (*ii*) *survival*, for environmental contexts relevant to individual fitness (Stubbersfield et al., 2015; Nairne et al., 2007; Otgaar & Smeets, 2010); (*iii*) *emotional*, that elicits strong positive or negative responses such as amusement or disgust (Heath et al., 2001; Eriksson & Coultas, 2014; Fessler et al., 2014; Stubbersfield et al., 2017); (*iv*) *moral*, regarding acceptable behavior and social norms (Heath et al., 2001; Baumard & Boyer, 2013; Stubbersfield et al., 2019); (*v*) *rational*, describing cause-and-effect connections (Bartlett, 1920; Glenn, 1980); and (*vi*) *counterintuintuive*, which defies ontological expectations in biological, physical, mental, and other domains (Barrett, 2008; Boyer & Ramble, 2001). Counterintuitive information can influence transmission in different ways: by themselves, counterintuitive elements can be more salient than other types of information (Boyer & Ramble, 2001); or, collectively, a minority of counterintuitive elements can lead to a minimally counterintuitive (“MCI”) bias that enhances overall recollection of a story (Stubbersfield & Tehrani, 2013; Norenzayan et al., 2006). The influence of rational content on cultural transmission was first examined in early experimental work by Bartlett (1920) but has not received much attention in the modern cultural evolution literature, though some related work has focused on causal reasoning and imitation (Bender & Beller, 2019; Berl & Hewlett, 2015; Buchsbaum et al., 2011; Horner & Whiten, 2005). We reintroduce rational information here as a type of content bias that could reasonably affect transmission dynamics. We crafted the narratives used in this study to resemble real-world creation stories in both form and the aforementioned types of content biases (see Methods). Real-world creation stories have evolved over many generations of transmission and selection, and therefore tend to contain biased content at high frequencies.

Beyond the types of information included in a story, learners are also sensitive to the identity and reputation of the storyteller. These model-based transmission biases include *prestige* (Henrich & Gil-White, 2001), *success* (Mesoudi, 2008), and *similarity bias* (Mahajan & Wynn, 2012; McElreath et al., 2003). In this study, we specifically examine prestige bias, which involves a preference to learn from individuals of high social position, reputation, and knowledge (Berl et al., 2020). Prestige is one of the most commonly cited transmission biases (Jiménez & Mesoudi, 2019), and has been implicated as one of the predominant forces in cultural change (Henrich & Gil-White, 2001; Henrich & Boyd, 2002; Henrich et al., 2015). However, the limited empirical work to date has shown mixed support regarding the extent to which the prestige of a model actually affects the adoption of a particular cultural variant or behavior (Atkisson et al., 2012; Chudek et al., 2012, 2016; Acerbi & Tehrani, 2018; Garfield et al., 2019; Jiménez & Mesoudi, 2020; Brand et al., 2020; see Jiménez & Mesoudi, 2019 for a recent review).

In our experiment, we use regional accents of speech as an experimental cue for prestige information. Studies in sociolinguistics—specifically micro-sociolinguistics, which focuses on the fine-scale components of social interaction—have shown that individual variants in spoken language (e.g. speech sounds, word choices) have social value (Ervin-Tripp, 1969; Lambert et al., 1960; Labov, 1966; Giles, 1970; Riches & Foddy, 1989; Bishop et al., 2005; Coupland & Bishop, 2007; Fuertes et al., 2012). Speakers constantly shift between the variants they know, including matching variants used in conversation, to affiliate themselves with others. Since unfamiliar linguistic variants are difficult to reproduce convincingly and so represent “hard-to-fake” signals (Cronk, 2005), variants become associated with membership in particular social groups, networks, or classes (Giles, 1970; Kahane, 1986; Kraus et al., 2019; Kroch, 1978). Clusters of speech variants become identifiable to others as accents, and over time they come to carry meanings, such as prestige, associated with those social groups. In two prior studies, we verified that different contemporary accents of English are perceived as having consistently divergent levels of prestige across listeners (for direct ratings of being “prestigious,” as well as for constituent items such as “reputable” and “powerful”) and that these prestige ratings are distinct from other positive attributes such as friendliness (Berl et al., 2020; Samarasinghe et al., 2019). These perceptions of accents are consistent with how prestige is understood in the cultural evolution literature, as symbolic markers of identity that act as cues for social preference and information quality (Henrich & Gil-White, 2001; Henrich, 2009; Mesoudi, 2016; Tamariz et al., 2011). Accent thus provides a rich methodological alternative to the use of attention, gaze, or group consensus, which are commonly used to operationalize prestige.

In this study, we address multiple gaps in the literature by explicitly quantifying learners’ recall of multiple distinct types of content, transmitted by speakers whose accents are associated with varying levels of prestige. By testing both content and model-based biases together in the experimental transmission of a narrative, we can examine the relative effects of a large suite of cognitive biases, factors that theory suggests shape the spread of information and the evolution of human culture. Due to the lack of solid theoretical predictions for the relative strengths of these diverse biases, we take a comparative approach that combines the influences of prestige bias, content biases, story effects, demographic effects, and individual variation under one modelling framework.

## Methods

### Experimental protocol

We administered our experiment through a custom web browser application on a secure university server. Participants first selected their location, which determined which of the locally-calibrated accent recordings they would hear. Participants were instructed that they would need to listen to a recording of the first story, which would play only once, and that they would be asked to recall the story in as much detail as possible.

After listening to a story, participants took part in a working memory distraction task based on the Visual Spatial Learning Test (Malec et al., 1991). This task involved playing three rounds of a game in which participants had to recall symbols and their positions on a grid. For the symbols, we used the 9 most dissimilar characters from the “BACS-1” artificial character set (Vidal et al., 2017). This distraction task took approximately 5 minutes to complete and also provided a measure of unbiased working memory, which we calculated as the number of cards placed on the grid that matched the positions displayed (regardless of the symbol), plus the number of cards placed on the grid that matched both the positions and symbols displayed, averaged across all three trials (equivalent to the Position Learning Index, or “PLI” score, of Malec et al., 1991).

On completing the distraction task, participants recorded their verbal recollection of the creation story. They were given the opportunity to pause and continue recording but were not allowed to return or re-record after advancing to the next task. This process, including the working memory distraction task, was then repeated for the second story and with the opposite-prestige accent. Story order and accent presentation, as well as combinations of these factors, were randomized across participants. Each participant heard one story in one accent condition and a separate story in the alternate accent condition (see Story production for details on stories).

After recording their recollections of both stories, participants listened to recordings of the *Comma Gets a Cure* passage (see Acknowledgements) read by the same speakers. To test that the different accents consistently indexed the expected differences in prestige, participants rated the speakers using the items for the Position-Reputation-Information (PRI) scale of individual prestige (Berl et al., 2020) as well as additional solidarity and dynamism domains. The PRI scale measures prestige as composed of three distinct but complementary domains, termed position (“an individual's relative place in the social hierarchy”), reputation (“social opinion and esteem”), and information (“value placed by society on the holders of wisdom, expertise, and learning”), and this three-factor structure was found to be robust across multiple samples and validation methods (Berl et al., 2020, p. 8). Solidarity, a measure of in-group similarity and trust, and dynamism, indicating enthusiasm or excitement, have been used in sociolinguistic studies as positive traits that are distinct from a status or prestige dimension (Fuertes et al., 2012), and so are used to isolate prestige effects from other attitudes toward accent.

Finally, participants completed a demographic questionnaire including standard demographic items as well as residence history and self-reported accents of English. We collected data pertaining to gender and ethnicity in line with ethical practices in research and guidelines from national statistical agencies (US Census Bureau, 2017; UK Statistics Authority, 2018).

### Participants

We recruited UK participants on the Prolific Academic platform (*n* = 96), and US participants on Amazon Mechanical Turk (*n* = 100) using TurkPrime (now CloudResearch; Litman et al., 2017). Participants were eligible to take part in this study if they: had not taken part in any previous studies by the researchers; had taken part in and had successfully completed over 95% of at least 100 studies on Prolific Academic or over 98% of at least 5,000 tasks on Amazon Mechanical Turk; and were native English speakers. We excluded data from 33 participants due to technical recording errors or external interference (e.g. a second person contributing to retelling).

### Story production

We selected creation stories as the narrative form to be used for this study because they are rich in the types of content proposed to be relevant to cultural transmission. They contain ideas that pertain to the origins of life, death, ecology, and human society, and are a familiar pattern cross-culturally for the transmission of knowledge, values, and meaning. Further, they have each individually been subject to many generations of transmission and transformation and are thus ideal for research on the products of cultural evolutionary processes.

In order to characterize the types and frequencies of content present in real-world creation stories and develop a template for use in developing the stories to be used in the experiment, we undertook a survey of creation stories using ethnographic data from the electronic Human Relations Area Files (eHRAF) World Cultures database (Human Relations Area Files). We conducted the survey by searching for “creation” (and its derivatives) or “origin” within texts indexed under the “mythology” subject code (#773). We performed the search in the Probability Sample Files (PSF) subset, which is a stratified random sample of 60 cultures, each representative of a different “culture area.” Our search returned 100 story extracts from 35 cultures, and from this we selected 4 texts for analysis on the basis of appropriate length (~300–800 words) and being written and shared by in-group authors rather than foreign ethnographers. The stories selected belonged to the A⋅chik Mande (referred to in eHRAF as “Garo”; Rongmuthu, 1960, pp. 261–263), Baganda (“Ganda”; Kaggwa, 1971, p. 1), Kainai (“Blackfoot”; Mountain Horse, 1979, pp. 111–113), and Kānaka Maoli (“Hawaiian”; Kamakau, 1968, p. 63) peoples. We also included the Genesis creation story (from the ancient Israelites), as presented in the New Revised Standard Version Bible (Coogan et al., 2010, Gen. 1.1–2.3). We coded the resulting 5 ethnographic creation stories for the presence of social, survival, emotional, moral, rational, and counterintuitive content biases. We examined each story, counting the number of occurrences of each bias at the sentence level, summing across the entire story, and calculating proportions of each bias by dividing by total word count. Definitions of these biases as used for coding and examples from the ethnographic creation stories are listed in Table 1.

**Table 1.** Definitions of content biases used in coding and example statements containing those content biases from selected ethnographic creation stories. Some quoted examples contain multiple instances of the indicated bias or additional biases beyond the indicated bias.

Content Bias	Definition	Example
**Social** **(Basic)**	*Interactions or relationships between individuals or groups*	“He began to shout and wave to attract the attention of the owners.” (Mountain Horse, 1979, p. 112)
**Social** **(Gossip)**	*Interactions or relationships between individuals or groups concerning third parties, or references to reputation or reputational costs, or the literal act of gossiping about other individuals*	“… God warned Kintu, ‘you must set off very early in the morning so that your brother Walumbe may not know where you have gone. Should you go with him, he will kill your children.’” (Kaggwa, 1971, p. 1)
**Survival**	*Explicit references to food, water, clothing, shelter, tools, predators, natural threats and disasters, seasonal cycles, reproduction, death, or disease in a survival context*	“Then God said, ‘Let the earth put forth vegetation: plants yielding seed, and fruit trees of every kind on earth that bear fruit with the seed in it.’” (Coogan et al., 2010, Gen. 1.11)
**Emotional (Positive)**	*Explicit displays or expressions of, or reference or reaction to, a positive emotional response beyond baseline, or an event that is expected to evoke a strong basic positive emotional response in the audience*	“In the time of their child Kapapaialaka… the god looked toward mankind with love, and men became the children of god.” (Kamakau, 1968, p. 63)
**Emotional (Negative)**	*Explicit displays or expressions of, or reference or reaction to, a negative emotional response beyond baseline, or an event that is expected to evoke a strong basic negative emotional response in the audience*	“At this Bamin Racha grew angry, chased the sowers far and wide, and at last overtook them at Ahratcha Rongbare where he threw them on the ground with their faces towards the sky, pierced their legs through, chained them together and let them go their way.” (Rongmuthu, 1960, p. 261)
**Moral**	*Deals with social norms, taboos, and values, deviation from social norms, and rewards for adherence or punishment for deviance*	“When Lihau'ula carried on his priestly work, certain foods were made kapu to women… It was made kapu for men and women to eat together. This was to separate from the worship of the god those who were unclean and defiled by blood…” (Kamakau, 1968, pp. 63–64)
**Rational**	*Concerning cause-and-effect relationships or employing causal reasoning*	“… if they should continue to indiscriminately sow the seeds of stones, rocks and cliffs over the whole surface of the earth, no one would be able to plough the fields with buffaloes or make dams for fishing and for storing water.” (Rongmuthu, 1960, p. 261)
**Counterintuitive**	*Violations of ontological properties of folk-biology, folk-psychology or folk-physics*	“Napi took this soil in his hands and commenced rolling it, and as he rolled the soil it multiplied and fell and blew and scattered from him until the present earth's surface was formed.” (Mountain Horse, 1979, p. 111)

For the experiments, we commissioned two written artificial creation stories (see Acknowledgements). We did this rather than using the ethnographic stories we sampled to ensure that our participants would all be equally unfamiliar with the stories, to have two different stories of comparable length and content for the two prestige conditions, and to avoid issues of cultural appropriation surrounding the use of stories from real societies. We recognize that stories artificially created to satisfy controls have not naturally evolved through cultural transmission and selection; however, the direct reproduction of stories from marginalized groups to further our own work while providing no real benefit to the members of those groups would be irresponsible from both an ethical and a scientific standpoint. To minimize this effect, a professional author wrote the stories—using the selected ethnographic stories as a template—and we aimed to preserve narrative flow in subsequent story revisions where we adjusted content frequencies. The first of the two stories, “Muki,” explains how the actions of a child abandoned by its parents shaped a rugged landscape and its varieties of life-forms. The second story, “Taka & Toro,” describes two jealous seafaring siblings and their competition over the friendship of the people they created. We carried out propositional analysis under the protocol established by Turner and Greene (1977) to define propositions (word clusters consisting of “a predicate plus a series of ordered arguments”; Mesoudi et al., 2006) as units of meaning within each story. We then iteratively edited the texts of these artificial creation stories to ensure the proportions of each content bias in each story matched one another, and also fell within 90% confidence intervals of the proportions seen in the coded ethnographic creation stories (see Supplementary Table S1). We tuned both stories to be approximately 850 words (Muki 887, Taka & Toro 835) and 270 propositions (Muki 265, Taka & Toro 273) to avoid ceiling effects for recall and to be of roughly equal complexity. Readability scores (based on number of syllables per word and sentence structure) for these artificial stories were roughly equivalent and used simpler language than the ethnographic stories they were modeled after (Flesch-Kincaid grade level: Muki 4.91, Taka & Toro 5.03, Ethnographic Mean 8.22 [90% CI: 6.42, 10.02]; Flesch reading ease: Muki 84.5, Taka & Toro 81.9, Ethnographic Mean 71.24 [90% CI: 62.24, 80.24]). The final versions of the two artificial creation stories, along with lists of their propositions and coded biases, can be found in the data repository (see Research transparency and reproducibility).

### Recordings

Using language accent to index prestige, we selected and recorded self-identified middle-aged white male speakers with high- and low-prestige accents calibrated for the participants’ locations telling the two stories (“Muki” and “Taka & Toro”). We selected the high- and low-prestige accents based on the results of a previous study (Samarasinghe et al., 2019). For both the UK and US participants, Received Pronunciation (“RP”) served as the high-prestige accent. For the UK sample, the low-prestige variant was a West Country accent, from South West England; and for the US sample, the low-prestige variant was an Inland South accent, spanning the southern Appalachian, Ozark, and Ouachita mountain ranges of the Southern United States. We standardized the recordings for volume and length (each 5 min, 19 s).

For an independent assessment of accent prestige, we also recorded our speakers reading the first paragraph of the *Comma Gets a Cure* passage. This passage contains words from Wells's lexical set, designed to highlight phonological variation between different accents of English (Wells, 1982). We presented these recordings (range 35 s to 39 s) to participants to confirm that their perceptions of the prestige of each speaker matched what was expected (see Experimental protocol).

### Data coding and transcription

We transcribed the audio files containing participants’ story recordings and coded each response for the presence or absence of each proposition from the original texts (see data repository for transcripts and coded recall data). Because we instructed participants that they did not need to recall the stories verbatim, we counted the presence of a proposition if the meaning remained consistent through different word choices or constructions (e.g. we accepted synonyms and did not penalize the order of recall). If an error in the retellings was carried forward in the story, we only marked it absent in the first instance. We only counted biased propositions as present if the retelling retained the biased element (e.g. social interaction, counterintuitive properties, etc.).

To assess intercoder reliability, a second researcher re-coded a subset of 33 recordings (representing approximately 10% of the sample). We found substantial agreement between the coders (Cohen's κ = 0.737, *p* < 0.01), and coders discussed any disagreements until reaching consensus on the final coded data.

### Data analysis

We used a set of generalized linear mixed models (GLMMs) to model the presence or absence of a particular proposition. Here, we tested the effects of eight different transmission biases by fitting a set of 58 candidate models that account for the potential effects of these biases in isolation and in combination with one another (Supplementary Table S2). For these models, the fixed effects we examined can be broken down into three categories of: 1) *story-based effects* (story, presentation order, and line number representing position in the story and quadratic line number representing primacy or recency effects); 2) *transmission biases* (prestige; and content biases: social, survival, positive emotional, negative emotional, moral, rational, and counterintuitive domain); and 3) *demographic effects* (country, gender, ethnicity, accent matching low-prestige speaker, childhood town size, childhood town matching region of low-prestige speaker, education, occupation, income, and working memory score). Though age was among the set of standard demographic variables that we collected from participants, we excluded it from our models because of a lack of any predictive theory for its effects on recall beyond its effects on working memory. The variable representing whether the participant's childhood town matched the region of the low-prestige speaker was included to test for increased recall due to accent familiarity or comprehensibility. We included random effects for participant and proposition in all models to capture the remaining variance from these sources. Variable descriptions and the full data set are available in the Repository.

Given the number of variables involved and the lack of prior predictive theory on the relative importance of simultaneous prestige and content biases to guide model selection, we took a modified stepwise approach to explore the space of candidate models by focusing on theoretically-based groupings of predictors, retaining features with high explanatory value, and ending by model-averaging coefficients (Burnham & Anderson, 2002). We first tested and compared the null and full models against models containing each fixed effect in isolation, groups of fixed effects in each of the three theory-based categories (listed above), and all possible combinations of the three categories (model numbers 1–31: Supplementary Table S2). We then fit a model containing only the fixed effects from the full model that were found to explain a significant proportion of the variation in recall (model 32), and again without income after finding that it led to overfitting (model 33). Finally, we tested a set of models that added each remaining individual fixed effect to model 33 in sequence to assess whether more refinement was needed (models 34–46), and again after including gender (models 47–58), which we found to improve fit in the previous step.

After all model fitting was complete, we compared models on the basis of each model's Akaike information criterion (AIC) score. Due to the lack of a single dominant model with a weight greater than 0.95, we averaged the parameters of all models according to their Akaike weights (Burnham & Anderson, 2002). As our main interest was in determining which factors had the strongest effects on the recall of propositions (Nakagawa & Freckleton, 2011), we determined full model-averaged parameter estimates using the “zero method” (Burnham & Anderson, 2002; Grueber et al., 2011). This substitutes a value of zero for parameter estimates and errors in models where the parameter does not appear and computes a weighted average for each parameter using the models’ Akaike weights.

We re-fit the full set of models using a continuous measure of the participants’ perceptions of the speaker's prestige—as factor scores from the PRI scale of individual prestige (Berl et al., 2020)—rather than the binary high-low prestige variable, for the subset of participants that provided this information (roughly two thirds of the full data set). Results were qualitatively similar; however, direct comparisons cannot be made due to these analyses being performed on a subset of the data with observations known to be “missing not at random” (“MNAR”; Rubin, 1976) from the experimental design.

We used the R statistical environment, version 3.5.1 (2018-07-02), for all analyses (R Core Team, 2018).

### Ethics statement

Prior approval for research protocols was obtained from the Colorado State University Institutional Review Board (protocol #014-16H) and the University of Bristol Faculty of Arts Human Research Ethics Committee (protocols #26561, #31041, and #38323). We obtained prior informed consent from all participants. We compensated all participants for their time at rates above local minimum wages based upon the time taken to complete the tasks (approximately $11.02 per hour for US participants and £7.92 per hour for UK participants).

Participant data were gathered via a secure web application hosted on a University of Bristol server. Participants were assigned a random unique ID and no directly identifying information was gathered from participants. Amazon Mechanical Turk Worker IDs were encrypted and anonymized through TurkPrime to prevent identifiability. Voice recordings and data were securely stored on the server, and were transferred using encryption to the server from participants’ devices and from the server to the researchers’ computers for analysis.

## Results

### Sample demographics

Our final sample consisted of 163 participants: 72 from the UK and 91 from the US. Subsamples from both countries were roughly equivalent in age (UK: *Median* = 35, *IQR* = 6; US: *Median* = 34, *IQR* = 14), with a skew toward women (UK: 63.9% women, 36.1% men; US: 53.8% women, 46.2% men; no non-binary genders), and more white participants than mixed or people of color (UK: 90.3% white, 0% mixed, 9.7% PoC; US: 78.0% white, 9.9% mixed, 12.1% PoC). Distributions of participants’ level of educational achievement, occupational categories, and income were similar between countries (see Supplementary Fig. S1). Participant age had a small negative correlation with working memory score (*r* = −0.239).

### Participants showed preferential recall of biased information

**Figure 1.**
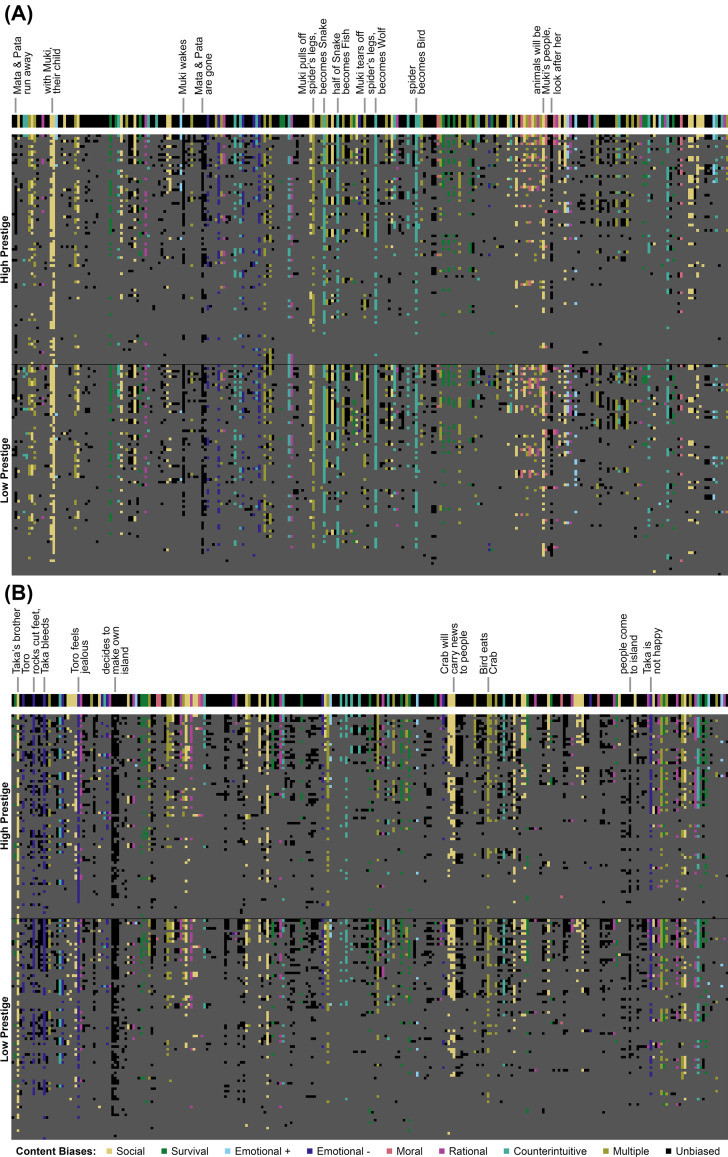
Color matrices of the presence or absence of propositions in recalled stories. Each row represents one participant's recall (*n* = 163 per panel, across high and low prestige conditions), sorted by hierarchical clustering to enhance visibility of patterns across participants. Each column is a proposition from the Muki (panel A) or Taka & Toro (panel B) artificial creation stories, from left to right in the order in which the propositions appeared in the stories. The thick line above each panel shows the full set of propositions contained in the story as originally told, with labels indicating propositions with exceptionally high recall (greater than 1.5 times the interquartile range: Tukey's definition of outliers). Within each panel, rows in the upper portion were read by a high-prestige speaker, while rows in the lower portion were read by a low-prestige speaker. Dark gray propositions were not recalled (absent). Recalled propositions (present) are each represented by a color that indicates the content biases they contained, as indicated in the legend at the bottom of the figure: *social* information is yellow, *survival* is green, *positive emotional* is light blue, *negative emotional* is dark purple, *moral* is pink, *rational* is magenta, all types of *counterintuitive* are teal, and propositions containing more than one bias are gold. Unbiased propositions, those that did not contain any biased information, are shown as black.

Of the 87,421 narrative propositions we presented in total, participants recalled 12,505 (14.3%) (Supplementary Table S3). We found a significant difference between the proportions of content types presented and the proportions of content types recalled (two-sided permutation test of independence: *z* = −2.037, *p* = 0.042), showing that participants recalled some types of biased information more frequently than other types, including unbiased information (i.e. propositions that did not contain any of the examined content biases; Fig. 1).

**Figure 2.**
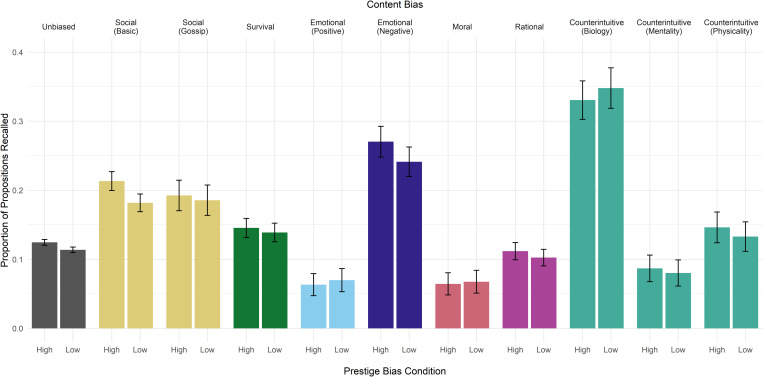
Mean proportion of propositions recalled from artificial creation stories by type of content bias and by speaker prestige. Error bars represent 95% confidence intervals. Of the total number of unbiased propositions or propositions containing a single content bias presented to participants, shown here, 13.8% were recalled (*n* = 10,864 propositions recalled out of 78,965 presented). Propositions containing more than one type of content bias (*n* = 1,641 propositions recalled out of 8,456 presented) are excluded from the figure to avoid depicting duplicate observations but are included in analyses.

Recall of each type of content bias ranged from a mean of 6.6% of the propositions presented (moral) to 33.9% (biological counterintuitive). In general, we observed small but non-significant differences in the recall of content biases in high- versus low-prestige speaker conditions (Fig. 2). However, pairwise chi-squared comparisons of proportions (corrected for false discovery rate using the Benjamini and Hochberg method; Supplementary Table S4) showed that prestige had a significant impact on the recall of unbiased information (*p* < 0.001) and basic social information (*p* = 0.001). Additionally, participants recalled unbiased information significantly less often than biased information under the same prestige condition (high-prestige unbiased versus survival *p* = 0.004, all others *p* < 0.001), except for positive emotional, moral, rational, and mental and physical counterintuitive information. Of these, positive emotional, moral, and mental counterintuitive information were recalled significantly less frequently than unbiased information in both prestige conditions (high-prestige unbiased versus mental *p* = 0.001, low-prestige unbiased versus mental *p* = 0.002, all others *p* < 0.001), while recall of rational and physical counterintuitive information were not significantly different from unbiased information in either prestige condition (all *p* > 0.06). When formal modeling is used to estimate the effects of these biases on recall while taking other factors into account (see below), we find that the biases of the propositions that were recalled significantly less frequently than unbiased information are not significant predictors of the probability of recall for a specific proposition.

### Content biases were more influential than prestige bias

To explain the variance in recall of specific propositions, we fit a total of 58 proposed models using maximum likelihood estimation (Supplementary Table S2). Models included different combinations of variables for story-based effects, the high or low prestige condition, the presence or absence of each content bias, and participant demographics (see Methods and Supplementary Table S2 for a full list). Eleven of the best-fitting models had a resulting ΔAIC score <2, indicating no single “best” model exists. The majority of best-fitting models included variables for story presentation order, for prestige, social, survival, negative emotional, and counterintuitive biases, and for gender and working memory (Supplementary Table S5).

**Figure 3.**
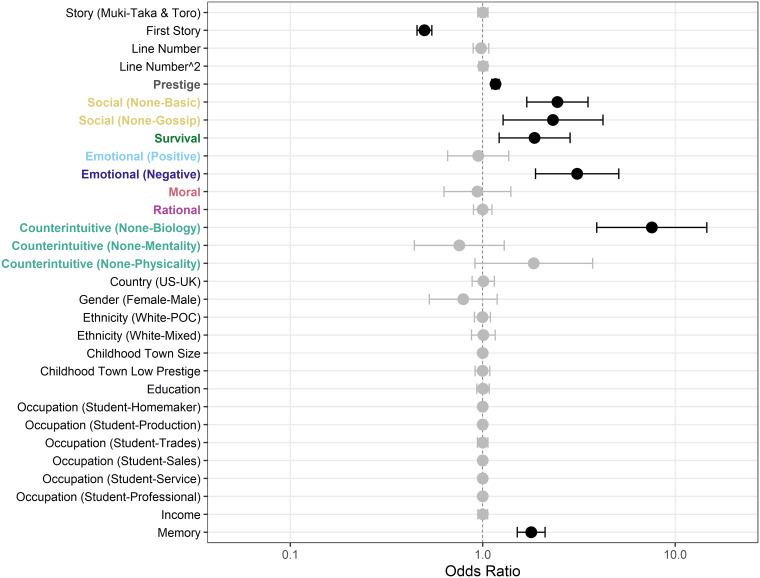
Forest plot of odds ratios from full model-averaged coefficients for fixed effects. Odds ratios and 95% confidence intervals are depicted such that variables for which confidence intervals do not overlap with 1 have a significant positive (above 1) or negative (below 1) effect on proposition recall (black), compared to variables that did not have a significant effect (gray). Binary and categorical variables are represented relative to the reference level (false/not present unless specified otherwise). For ordinal variables (childhood town size, education, and income), only linear contrasts are shown.

Our results (Fig. 3; Supplementary Table S6) show that the transmission biases with the greatest effect on recall were, in descending order: counterintuitive (biological violations), negative emotional, social, survival, and prestige biases. All other biases had negligible effects according to their model-averaged coefficients and confidence intervals and their relative variable importance values. Prestige, while significant, was the weakest of the transmission biases that had significant effects, with an odds ratio of 1.164 (95% CI [1.113, 1.217]) compared to the next lowest, survival, with 1.858 (95% CI [1.216, 2.841]) and to the strongest effect, biological counterintuitive, with 7.558 (95% CI [3.913, 14.597]). Notably, some biases that were recalled less frequently than unbiased information in terms of overall proportions (i.e. positive emotional, moral, and mental counterintuitive information; Fig. 2) did not show significant effects in predicting the probability of recall for a specific proposition. For story effects, participants had better recall for the second story they were presented, regardless of which story it was. The position of propositions within the story, represented by line number and quadratic line number, had no effect on recall. For demographic variables, only working memory had a significant positive effect.

To address a reviewer's suggestion that there could be a potential effect of accent similarity between participants and speakers beyond the effect of prestige alone, we fit an additional version of our best-fitting model (model 41), adding a fixed effect of childhood location (“childhood town low prestige”) and an interaction term between prestige condition and childhood location. The fitted model did not show a significant interaction effect between prestige and childhood location (*p* = 0.474), nor a significant main effect of childhood location (*p* = 0.829). The ΔAIC between this model and the best-fitting model was 3.40, and its AIC value was 1.50 greater than an identical model that only excluded the interaction effect (model 44). As this model constituted a *post hoc* evaluation of the potential similarity effect, we did not include it in the model-averaging procedure.

### Transmission biases explain little variance in recall

The set of best-fitting models (ΔAIC < 2) had relatively high mean conditional *R^2^_GLMM_* values at 0.524 (*SD* < 0.001), but a lower marginal *R^2^_GLMM_* at 0.106 (*SD* = 0.002). The difference between the two values represents the proportion of the variance explained by the random effects of the model, which were the participant ID (i.e. individual differences) and proposition ID. Comparisons of the lowest-AIC model with ones excluding either random effect using likelihood-ratio tests were both significant (participantID *X^2^* [1] = 6526.1; proposition *X^2^* [1] = 9728.2; both *p* << 0.001), indicating that the individual participant and proposition effects were both influential. These results tell us that there is a great deal of variance in our responses that is not accounted for by the transmission biases and other fixed effects included in the models. Further, they indicate that this unexplained variance exists both among the participants and within the content of the stories.

## Discussion

### Prestige bias has a minor effect on transmission

We found significant positive effects for prestige, social, survival, negative emotional, and biological counterintuitive biases on recall (see Fig. 3; Supplementary Table S6). Despite the prominence of prestige-biased transmission in the cultural evolution literature, prestige bias as proxied by accent had the smallest effect on transmission, increasing the likelihood of a proposition's recall by only 15%. One possible explanation for the secondary importance of prestige concerns the nature of the narratives transmitted. Transmission biases can lead to the development of group markers and in-group cooperation (Boyd et al., 2011; Boyd & Richerson, 2009; McElreath et al., 2003), and creation stories are representative of a shared group identity (Smith et al., 2017). If the audience does not perceive some cultural relationship between themselves and the storyteller or narrative, prestige may be a less pertinent cue for social learning. Prior work suggests that prestige often exists as an in-group hierarchy with less relevance to outgroup individuals (Halevy et al., 2012; Henrich et al., 2015). A second possibility for the relatively small size of our prestige effect is that, while we established that accent can be a reliable proxy for prestige (Samarasinghe et al., 2019), accent may not be perceived as a good indicator of prestige in the specific context of a transmission event. Future work should contrast and combine accent with other signifiers of prestige to understand their respective effects on the transmission process (Tamariz et al., 2011).

Assuming that shared identity could be a factor mediating the efficacy of prestige bias—in effect, a similarity bias (McElreath et al., 2003)—we examined links between participant and storyteller demographics. From this argument, participants should better recall a narrative read by a speaker whose accent they could identify with personally. However, our results show no effect on recall from matching participants’ childhood location with the region of the low-prestige speaker's accent. The formal inclusion of an interaction effect between prestige and childhood location failed to improve model fit compared to the model without the interaction term. We also addressed similarity bias through the standardization of speaker demographics and the inclusion of participants’ demographics in the models, finding no evidence that similarity between participants’ identities and those of the speakers substantially affected recall. These results also suggest there was no noteworthy effect of poor clarity or comprehensibility from accents that would be less familiar to a participant given their background.

Our finding that the effect of prestige on recall was small is consistent with the results of some previous experimental studies that have either failed to find any significant effect from prestige bias or have found the effect to be smaller than predicted (Chudek et al., 2016; Garfield et al., 2019; Jiménez & Mesoudi, 2020), and with an experimental study that compared prestige and content biases (Verpooten & Dewitte, 2017). In that study, the authors found that all participants’ appreciation of an art piece was affected by the aesthetic content of the work, but that only art professionals and experts were affected by context of the work's prestige as part of a prominent museum collection, pointing to the domain specificity of prestige in this instance. These studies and our own suggest a relationship between the source of a model's prestige and the domain of knowledge being transmitted that can lead to differential prestige effects depending on how closely the learner perceives these factors to be aligned. In our case, with the use of accent, it is possible that traditionally low-prestige accents associated with rural areas and blue-collar work, for instance, could actually be preferred for certain types of information and domains of knowledge.

### Prestige is unconsciously employed as a secondary bias

Another potential explanation for the low importance of prestige in determining recall is that participants may adjust their social learning strategies depending on which biases are present in different parts of the narrative (McElreath et al., 2008; Morgan et al., 2012; Rendell et al., 2011). When content biases were present, prestige had less relative influence on recall, but participants tended to recall unbiased propositions more frequently when the narrative was told by a speaker with a high-prestige accent (Fig. 2).

The finding that prestige takes a secondary role to content supports the conclusions of a second prior experimental study that compared prestige and content (Acerbi & Tehrani, 2018). In this study, the authors found that the effects of prestige were minimal compared to content effects (characterized as “inspiration” or general likability rather than specific biases) when rating their preference for quotations from famous or unknown authors. Taken together with previous experimental work, our results demonstrate the importance of content biases in directing cultural transmission. These content cues can be more nuanced than general context-based copying rules such as prestige, but our results show that content biases can take a primary role over prestige, which had a conditional effect. Future studies could explore how the relative influence of content versus model- and context-based biases may vary across different sociocultural contexts and across different types of information (such as the transfer of highly structured skills, like stone tool knapping, where there may be less opportunity for variance in content), and the potential interactive effects between different forms of biases (for example, one character feeding or caring for another may encode both social and survival information, and be more or less salient than either type of content individually).

### Content biases have distinct effects

We found that the effects of content types on information transmission varied widely (Fig. 3). Prior theory would suggest a greater attention to “gossip” over basic social interactions (Dunbar, 1993, 1996), but we found no significant difference between the two in our results (Fig. 2), which is consistent with previous work (Mesoudi et al., 2006). In our study, gossip was qualified by the presence of third parties in social interactions rather than the emotional intensity of the interactions (Mesoudi et al., 2006), and we coded specific propositions with social interaction as either basic or gossip rather than entire passages. Any advantageous impact of social “gossip” on transmission also may have been tempered by the cognitive load of processing multiple levels of theory of mind in these interactions (Dunbar, 1998, 2004).

Our results add support to multiple prior empirical studies that found strong positive effects on transmission for survival information (Nairne et al., 2007; Nairne & Pandeirada, 2010; Otgaar & Smeets, 2010; Stubbersfield et al., 2015), and for negative emotional information but not positive emotional information (Bebbington et al., 2017; Heath et al., 2001; Kensinger & Corkin, 2003). Negative emotional information was found to be one of the most powerful biases in our stories (Fig. 2). As negative information arouses strong emotional responses such as fear, disgust, and anger, some theorize that humans evolved broad cognitive domains receptive to negative information as a survival response to predators and toxic food sources (Al-Shawaf et al., 2016; Al-Shawaf & Lewis, 2017; Barrett, 2015; Boyer & Barrett, 2015; Tooby & Cosmides, 2008), which may explain why both survival and negative emotional information are particularly salient.

We did not find evidence to support effects from moral, rational, or most counterintuitive information on transmission. Moral and mental counterintuitive information (as well as positive emotional, above) were actually recalled less often than unbiased information (Fig. 2), though not enough to lead to negative odds ratios when accounting for other variables (Fig. 3). However, there have been few prior tests of these biases within an experimental transmission paradigm. For instance, previous evidence of a bias for “rational” or causal information in this context has been anecdotal (Bartlett, 1920) and merits further investigation. The transmission of rational information relies upon the retention of a predicate. We defined successful transmission of rational information as requiring the retention of the subordinating conjunction (“because,” “so that,” “when,” etc.; i.e. the proposition being coded as having rational content), which may explain the lack of an effect. Hence, rational bias may have instead had a proximity effect on the recall of surrounding information, without being recalled itself, that was not detected by our present analyses.

For moral information, according to social norm theory, individuals should be expected to retain and transmit moral information depending, firstly, on the strength of the social norm and, secondly, on the extent to which they identify with the social group to which it applies (Cialdini & Trost, 1998; McDonald & Crandall, 2015). That participants did not recall moral information is less surprising if they recognized that the creation stories did not describe their own society's origins or rules of accepted behavior. Stubbersfield et al. (2019) found that transmission was higher for moral information only when it was related to good or virtuous acts. We did not distinguish between moral categories in our coding, but the moral information in the creation stories we sampled and those we created tended to be proscriptive, so this could be an additional explanation for our findings.

### Narrative structural features may aid transmission

To the best of our knowledge, no existing theory addresses why particular counterintuitive domains should be recalled more or less frequently than others. However, our data demonstrate that biological counterintuitive information was significantly more likely to be transmitted than other types. This result may not necessarily be due to the bias *per se*, but rather could be a consequence of narrative construction. Many of the biological counterintuitive propositions in our stories were repetitive in structure (for example, in the “Muki” story, spiders were transformed into other animals four times in sequence), and recollection may be affected by what Jakobson (1960) called the “poetic function” of language (Waugh, 1980), or the artistic quality of the message itself. In our study design, we credit a causal role to linguistic factors in social learning through our use of accent-based prestige; however, narrative theory itself remains a rich and largely untapped resource in cultural evolutionary accounts of information transmission (Rivkin & Ryan, 2004).

For stories to be impactful, the content must engage the audience (Busselle & Bilandzic, 2009; Duranti, 1986; Graesser et al., 2002) and compete for space in working memory (Graesser et al., 2003; Kormos & Trebits, 2011; Montgomery et al., 2009; Ward et al., 2016). To this end, stories (and their tellers) employ a suite of features to enhance their salience, including elements that evoke emotional arousal (Andringa, 1996; Hänninen, 2007; Komeda et al., 2009; Benelli et al., 2012) and the use of familiar narrative devices such as rich encoding and repetition (Genter, 1976; Thorndyke, 1977). As such, multiple factors influence the success of story transmission and our results demonstrate that transmission biases alone do not capture the full variation.

### Implications for the understanding of transmission

The overall fit of our model is high (*R^2^_GLMMc_* = 0.524), but fixed effects only explain a small portion of the variation in recall (*R^2^_GLMMm_* = 0.106). One possible explanation for this result is that some as-yet unidentified biases exist in the characteristics of the models or in the content of the stories, and these drove the variation in proposition transmission. However, our methodological approach included every type of content bias supported in the literature, and we could not test the remaining well-documented types of model-based and context biases, such as conformity bias (Efferson et al., 2008) and success bias (Baldini, 2012), because they did not apply to the isolated one-to-one transmission context of our experiment. In the future, if additional content biases are identified in the literature, it would be possible for researchers to re-code our data to test them.

Instead, the substantial explanatory power of the random effects in our models may represent the noise of individual variation: a trade-off for real-world validity through variation in experimental circumstances. Our methodological approach did not allow us to control the testing environments, including levels of distraction, participants’ levels of attention, or participants’ personal short- and long-term histories. In this way, the experiment mimics real-world cultural transmission, which tends to be filled with random noise that can lead to low fidelity in one-off transmission events (Efferson et al., 2007; Strimling et al., 2009). Much debate exists regarding the degree of transmission fidelity required for cumulative culture. Some argue that high-fidelity transmission is required (Tomasello et al., 1993; Tennie et al., 2009; Lewis & Laland, 2012; Dean et al., 2014; Caldwell et al., 2018), while others counter that low-fidelity transmission is sufficient (Sperber, 1996; Sasaki & Biro, 2017; Zwirner & Thornton, 2015; McElreath et al., 2018; Miton & Charbonneau, 2018; Truskanov & Prat, 2018; Miu et al., 2018) and that weak biases can be amplified over repeated rounds of transmission to create strong universal patterns (Strimling et al., 2009; Kirby et al., 2007; Thompson et al., 2016). We found that participants’ responses to identical stimuli varied significantly, and that transmission fidelity was often low compared to previous studies (e.g. Mesoudi et al., 2006). Participants knew they would need to retain and recite the information but, on average, they recalled only 14.3% of the propositions presented. However, in the context of a single-shot experimental transmission event where payoff is not dependent on recall, participants have no real incentive to retain information, and these stories were intentionally of considerable length (to avoid ceiling effects) and posed a substantial challenge for working memory. Furthermore, whereas we presented each story only once, repeated exposure to a story increases comprehension (Dennis & Walter, 1995), and narratives that particularly define a group—such as creation stories—are often told multiple times (Norrick, 2007) or are collaborative, with opportunities for audience engagement that allow group members to transform and take ownership of the narrative (Lawrence & Thomas, 1999; Norrick, 1997). On the other hand, the substantial amount of individual variation observed across our transmission events could suggest that transmission is inherently a highly heterogeneous process, and may require the development of complex and nuanced models to tease apart individual effects on micro-scale transmission processes and the population-level cultural evolutionary dynamics that arise from them.

Our methodological and analytical framework provides a template for future tests of the simultaneous effects of content biases and model-based biases, and for other context-based biases. We have performed an experimental test of the relative effects of multiple types of cultural transmission biases presented within a realistic package of narrative information, while incorporating linguistic factors that have been underutilized in the cultural evolution literature. Although we found that prestige was the least important bias among those that had measurable effects, it was still a significant factor in participants’ choices of what information to retain and recall, especially for information lacking any internal biases. Our results suggest that the prominent role of prestige-biased transmission models in cultural evolution studies should be scrutinized more heavily and qualified by the presence or absence of other biases, which may have stronger effects under certain conditions. Given varied definitions and operationalizations of prestige, we propose that future work should examine the use of binary versus continuous measures of prestige (e.g. the PRI scale: Berl et al., 2020) and first-order versus second-order prestige cues (Jiménez & Mesoudi, 2019). Studies are also needed to test the effects of prestige and other biases across different transmission mechanisms and pathways, for example by comparing results from an experimental design in which participants are forced to choose between alternative models against a design in which participants are exposed to all models and asked to recall or copy each of them (as here). Additionally, recall may be necessary but not sufficient for further transmission of a cultural variant if it is not expressed through behavior or if the variant is not learned and demonstrated by a sufficiently influential model.

The experimental framework presented here also sets the stage for future research to test other longstanding questions in cultural evolution, such as: which biases are necessary or sufficient for the development of cumulative culture (Tomasello et al., 1993), which conditions cause learners to favor one type of bias over another (Rendell et al., 2011), whether and how the effects of specific biases differ cross-culturally (Efferson et al., 2007; Mesoudi et al., 2014; Eriksson et al., 2016; Leeuwen et al., 2018), how micro-level transmission processes lead to macro-level cultural change (Mesoudi et al., 2006; Schwartz & Mead, 1961), and how we can identify the bias or biases responsible for a *post hoc* distribution of traits (Kandler & Powell, 2015). The results of this study go beyond academic discourse in cultural evolution to impact other disciplines that rely on the theory and application of communication as a means of disseminating information and motivating behavior change, including education, biological and cultural conservation, public health, political science, and marketing. Storytelling persists as a powerful and enduring cultural tool, dense in information critical for survival and social cohesion, and utilized across the world to share knowledge and shape the diversity of human culture.

## Supplementary material

For supplementary material accompanying this paper visit https://doi.org/10.1017/ehs.2021.37. click here to view supplementary material

## Research Transparency and Reproducibility

All data and R scripts used for analysis are in a public OSF project (“the data repository”), titled “Cultural Transmission,” available at: https://osf.io/72v3f/. Source code and texts for the custom SurveyJS web application used for data collection are in a public GitHub repository, available at: https://github.com/seannyD/StoryTransmission.
