# Supplementary material for: Prestige and content biases together shape the cultural transmission of narratives
Source: Evol Hum Sci. 2021 Jul 29;3:e42. doi: 10.1017/ehs.2021.37 (PMC10427335; doi:10.1017/ehs.2021.37)
Supplement: Supplementary file 1 [file S2513843X21000372sup001.docx]

Supplementary Material for

**Prestige and content biases together shape the cultural transmission of narratives**

Richard E.W. Berl^1^*^†^, Alarna N. Samarasinghe^2†^, Seán G. Roberts^2,3^, Fiona M. Jordan^2,4^, Michael C. Gavin^1,4^

^1^ Department of Human Dimensions of Natural Resources, Colorado State University, Fort Collins, CO 80523-1480, USA

^2^ Department of Anthropology and Archaeology, University of Bristol, Bristol, United Kingdom

^3^ School of English, Communication and Philosophy, Cardiff University, Cardiff, United Kingdom

^4^ Max Planck Institute for the Science of Human History, Jena, Germany

* Corresponding author: Richard E.W. Berl, rewberl@colostate.edu

^†^ R.E.W.B. and A.N.S. contributed equally to this work and are co-first authors.

**Supplementary Tables**

**Supplementary Table S1. Number of instances of biased content in ethnographic creation stories and artificial creation stories, divided by word count.**

| Content Bias | 90% CI for ethnographic  creation stories | Muki | Taka & Toro |
| --- | --- | --- | --- |
| Social (Basic) | [0.707, 4.748] | 3.833 | 3.952 |
| Social (Gossip) | [0.000, 1.326] | 1.240 | 1.198 |
| Survival | [1.152, 2.718] | 2.480 | 2.635 |
| Emotional (Positive) | [0.009, 1.088] | 1.015 | 1.078 |
| Emotional (Negative) | [0.000, 2.137] | 1.804 | 1.796 |
| Moral | [0.466, 1.679] | 1.015 | 0.958 |
| Rational | [1.269, 2.894] | 2.255 | 2.515 |
| Counterintuitive | [0.216, 0.583] | 0.338 | 0.359 |

**Supplementary Table S2. Full set of candidate generalized linear mixed models tested.**

| Number | Name | story | first story | line | line^2 | prestige | social | survival | emotional  positive | emotional  negative | moral | rational | counterintuitive  domain | country | gender | ethnicity | childhood town  size | childhood town  low prestige | education | occupation | income | memory |
| --- | --- | --- | --- | --- | --- | --- | --- | --- | --- | --- | --- | --- | --- | --- | --- | --- | --- | --- | --- | --- | --- | --- |
| 1 | Null |  |  |  |  |  |  |  |  |  |  |  |  |  |  |  |  |  |  |  |  |  |
| 2 | Full | + | + | + | + | + | + | + | + | + | + | + | + | + | + | + | + | + | + | + | + | + |
| 3 | Story effects | + | + | + | + |  |  |  |  |  |  |  |  |  |  |  |  |  |  |  |  |  |
| 4 | Story | + |  |  |  |  |  |  |  |  |  |  |  |  |  |  |  |  |  |  |  |  |
| 5 | First story |  | + |  |  |  |  |  |  |  |  |  |  |  |  |  |  |  |  |  |  |  |
| 6 | Line number |  |  | + |  |  |  |  |  |  |  |  |  |  |  |  |  |  |  |  |  |  |
| 7 | Quadratic line number |  |  | + | + |  |  |  |  |  |  |  |  |  |  |  |  |  |  |  |  |  |
| 8 | Biases |  |  |  |  | + | + | + | + | + | + | + | + |  |  |  |  |  |  |  |  |  |
| 9 | Prestige |  |  |  |  | + |  |  |  |  |  |  |  |  |  |  |  |  |  |  |  |  |
| 10 | Content |  |  |  |  |  | + | + | + | + | + | + | + |  |  |  |  |  |  |  |  |  |
| 11 | Social |  |  |  |  |  | + |  |  |  |  |  |  |  |  |  |  |  |  |  |  |  |
| 12 | Survival |  |  |  |  |  |  | + |  |  |  |  |  |  |  |  |  |  |  |  |  |  |
| 13 | Emotional |  |  |  |  |  |  |  | + | + |  |  |  |  |  |  |  |  |  |  |  |  |
| 14 | Emotional (positive) |  |  |  |  |  |  |  | + |  |  |  |  |  |  |  |  |  |  |  |  |  |
| 15 | Emotional (negative) |  |  |  |  |  |  |  |  | + |  |  |  |  |  |  |  |  |  |  |  |  |
| 16 | Moral |  |  |  |  |  |  |  |  |  | + |  |  |  |  |  |  |  |  |  |  |  |
| 17 | Rational |  |  |  |  |  |  |  |  |  |  | + |  |  |  |  |  |  |  |  |  |  |
| 18 | Counterintuitive |  |  |  |  |  |  |  |  |  |  |  | + |  |  |  |  |  |  |  |  |  |
| 19 | Demographics |  |  |  |  |  |  |  |  |  |  |  |  | + | + | + | + | + | + | + | + | + |
| 20 | Country |  |  |  |  |  |  |  |  |  |  |  |  | + |  |  |  |  |  |  |  |  |
| 21 | Gender |  |  |  |  |  |  |  |  |  |  |  |  |  | + |  |  |  |  |  |  |  |
| 22 | Ethnicity |  |  |  |  |  |  |  |  |  |  |  |  |  |  | + |  |  |  |  |  |  |
| 23 | Town size |  |  |  |  |  |  |  |  |  |  |  |  |  |  |  | + |  |  |  |  |  |
| 24 | Town low prestige |  |  |  |  |  |  |  |  |  |  |  |  |  |  |  |  | + |  |  |  |  |
| 25 | Education |  |  |  |  |  |  |  |  |  |  |  |  |  |  |  |  |  | + |  |  |  |
| 26 | Occupation |  |  |  |  |  |  |  |  |  |  |  |  |  |  |  |  |  |  | + |  |  |
| 27 | Income |  |  |  |  |  |  |  |  |  |  |  |  |  |  |  |  |  |  |  | + |  |
| 28 | Memory |  |  |  |  |  |  |  |  |  |  |  |  |  |  |  |  |  |  |  |  | + |
| 29 | Story effects and biases | + | + | + | + | + | + | + | + | + | + | + | + |  |  |  |  |  |  |  |  |  |
| 30 | Story effects and demographics | + | + | + | + |  |  |  |  |  |  |  |  | + | + | + | + | + | + | + | + | + |
| 31 | Biases and demographics |  |  |  |  | + | + | + | + | + | + | + | + | + | + | + | + | + | + | + | + | + |
| 32 | Significant variables from full model |  | + |  |  | + | + | + |  | + |  |  | + |  |  |  |  |  |  |  | + | + |
| 33 | Significant variables from full model without income (“A”) |  | + |  |  | + | + | + |  | + |  |  | + |  |  |  |  |  |  |  |  | + |
| 34 | A with story | + | + |  |  | + | + | + |  | + |  |  | + |  |  |  |  |  |  |  |  | + |
| 35 | A with line number |  | + | + |  | + | + | + |  | + |  |  | + |  |  |  |  |  |  |  |  | + |
| 36 | A with quadratic line number |  | + | + | + | + | + | + |  | + |  |  | + |  |  |  |  |  |  |  |  | + |
| 37 | A with positive emotional |  | + |  |  | + | + | + | + | + |  |  | + |  |  |  |  |  |  |  |  | + |
| 38 | A with moral |  | + |  |  | + | + | + |  | + | + |  | + |  |  |  |  |  |  |  |  | + |
| 39 | A with rational |  | + |  |  | + | + | + |  | + |  | + | + |  |  |  |  |  |  |  |  | + |
| 40 | A with country |  | + |  |  | + | + | + |  | + |  |  | + | + |  |  |  |  |  |  |  | + |
| 41 | A with gender |  | + |  |  | + | + | + |  | + |  |  | + |  | + |  |  |  |  |  |  | + |
| 42 | A with ethnicity |  | + |  |  | + | + | + |  | + |  |  | + |  |  | + |  |  |  |  |  | + |
| 43 | A with town size |  | + |  |  | + | + | + |  | + |  |  | + |  |  |  | + |  |  |  |  | + |
| 44 | A with town low prestige |  | + |  |  | + | + | + |  | + |  |  | + |  |  |  |  | + |  |  |  | + |
| 45 | A with education |  | + |  |  | + | + | + |  | + |  |  | + |  |  |  |  |  | + |  |  | + |
| 46 | A with occupation |  | + |  |  | + | + | + |  | + |  |  | + |  |  |  |  |  |  | + |  | + |
| 47 | A with gender and story | + | + |  |  | + | + | + |  | + |  |  | + |  | + |  |  |  |  |  |  | + |
| 48 | A with gender and line number |  | + | + |  | + | + | + |  | + |  |  | + |  | + |  |  |  |  |  |  | + |
| 49 | A with gender and quadratic line number |  | + | + | + | + | + | + |  | + |  |  | + |  | + |  |  |  |  |  |  | + |
| 50 | A with gender and positive emotional |  | + |  |  | + | + | + | + | + |  |  | + |  | + |  |  |  |  |  |  | + |
| 51 | A with gender and moral |  | + |  |  | + | + | + |  | + | + |  | + |  | + |  |  |  |  |  |  | + |
| 52 | A with gender and rational |  | + |  |  | + | + | + |  | + |  | + | + |  | + |  |  |  |  |  |  | + |
| 53 | A with gender and country |  | + |  |  | + | + | + |  | + |  |  | + | + | + |  |  |  |  |  |  | + |
| 54 | A with gender and ethnicity |  | + |  |  | + | + | + |  | + |  |  | + |  | + | + |  |  |  |  |  | + |
| 55 | A with gender and town size |  | + |  |  | + | + | + |  | + |  |  | + |  | + |  | + |  |  |  |  | + |
| 56 | A with gender and town low prestige |  | + |  |  | + | + | + |  | + |  |  | + |  | + |  |  | + |  |  |  | + |
| 57 | A with gender and education |  | + |  |  | + | + | + |  | + |  |  | + |  | + |  |  |  | + |  |  | + |
| 58 | A with gender and occupation |  | + |  |  | + | + | + |  | + |  |  | + |  | + |  |  |  |  | + |  | + |

A plus sign indicates that a variable was included in the specified model. Random effects of participant and proposition number were included in all models.

**Supplementary Table S3. Three-way table of biases present in artificial story propositions.**

|  |  | **Social** | **Survival** | **Emotional (Positive)** | **Emotional (Negative)** | **Moral** | **Rational** | **Counter-intuitive** | **Unbiased** |
| --- | --- | --- | --- | --- | --- | --- | --- | --- | --- |
| **Social** | Presented | 58  (10.8%) | 5  (0.9%) | 4  (0.7%) | 4  (0.7%) | 2  (0.4%) |  | 14  (2.6%) |  |
|  | Recalled | 1841  (14.7%) | 269  (2.2%) | 85  (0.7%) | 246  (2.0%) | 70  (0.6%) |  | 270  (2.2%) |  |
| **Survival** | Presented |  | 31  (5.8%) |  | 2  (0.4%) |  | 3  (0.6%) | 2  (0.4%) |  |
|  | Recalled |  | 716  (5.7%) |  | 42  (0.3%) |  | 80  (0.6%) | 103  (0.8%) |  |
| **Emotional (Positive)** | Presented |  |  | 11  (2.0%) |  | 1  (0.2%) |  | 2  (0.4%) |  |
|  | Recalled |  |  | 119  (1.0%) |  | 4  (< 0.1%) |  | 41  (0.3%) |  |
| **Emotional (Negative)** | Presented |  |  |  | 19  (3.5%) | 1  (0.2%) | 1  (0.2%) | 3  (0.6%) |  |
|  | Recalled |  |  |  | 789  (6.3%) | 20  (0.2%) | 6  (< 0.1%) | 131  (1.0%) |  |
| **Moral** | Presented |  |  |  |  | 11  (2.0%) | 2  (0.4%) |  |  |
|  | Recalled |  |  |  |  | 118  (0.9%) | 38  (0.3%) |  |  |
| **Rational** | Presented |  |  |  |  |  | 30  (5.6%) | 5  (0.9%) |  |
|  | Recalled |  |  |  |  |  | 522  (4.2%) | 185  (1.5%) |  |
| **Counter-intuitive** | Presented |  |  |  |  |  |  | 35  (6.5%) |  |
|  | Recalled |  |  |  |  |  |  | 1126  (9.0%) |  |
| **Unbiased** | Presented |  |  |  |  |  |  |  | 291  (54.1%) |
|  | Recalled |  |  |  |  |  |  |  | 5633  (45.0%) |

The first row within each bias gives the number of propositions (and percentage of the total) presented to **each** participant across both stories (N = 538 propositions per participant), while the second row within each bias gives the number of propositions (and percentage of the total) recalled across **all** participants (N = 12,505 propositions total). Columns indicate an additional type of bias present in the same proposition, such that numbers on the diagonal (e.g. Social-Social) represent propositions with only the single indicated bias, while off-diagonals (e.g. Social-Moral) represent propositions that contained both indicated biases. The lower triangle of the matrix is symmetric and so is not shown, for clarity. One proposition in the original stories (at 0.2% of the total) contained three biases (Social, Survival, and Negative Emotional) and this proposition was recalled 51 times (0.4%). This proposition is not depicted in the table but was included in analyses and the calculated percentages in the table reflect its inclusion. The final diagonal cells (in the bottom right corner) indicate unbiased propositions, or those that did not contain any of the content biases examined.

**Supplementary Table S4. Results of pairwise comparisons of proportions for content bias type and prestige speaker condition.**

|  |  | **Social (Basic)** | | **Social (Gossip)** | | **Survival** | | **Emotional (Positive)** | | **Emotional (Negative)** | | **Moral** | | **Rational** | | **Counterintuitive** | | **Unbiased** |
| --- | --- | --- | --- | --- | --- | --- | --- | --- | --- | --- | --- | --- | --- | --- | --- | --- | --- | --- |
|  |  | High | Low | High | Low | High | Low | High | Low | High | Low | High | Low | High | Low | High | Low | High |
| **Social (Basic)** | High |  |  |  |  |  |  |  |  |  |  |  |  |  |  |  |  |  |
|  | Low | **0.001** |  |  |  |  |  |  |  |  |  |  |  |  |  |  |  |  |
| **Social (Gossip)** | High | 0.149 | 0.466 |  |  |  |  |  |  |  |  |  |  |  |  |  |  |  |
|  | Low | 0.053 | 0.816 | 0.729 |  |  |  |  |  |  |  |  |  |  |  |  |  |  |
| **Survival** | High | **0.000** | **0.000** | **0.000** | **0.003** |  |  |  |  |  |  |  |  |  |  |  |  |  |
|  | Low | **0.000** | **0.000** | **0.000** | **0.000** | 0.557 |  |  |  |  |  |  |  |  |  |  |  |  |
| **Emotional (Positive)** | High | **0.000** | **0.000** | **0.000** | **0.000** | **0.000** | **0.000** |  |  |  |  |  |  |  |  |  |  |  |
|  | Low | **0.000** | **0.000** | **0.000** | **0.000** | **0.000** | **0.000** | 0.681 |  |  |  |  |  |  |  |  |  |  |
| **Emotional (Negative)** | High | **0.000** | **0.000** | **0.000** | **0.000** | **0.000** | **0.000** | **0.000** | **0.000** |  |  |  |  |  |  |  |  |  |
|  | Low | **0.036** | **0.000** | **0.003** | **0.001** | **0.000** | **0.000** | **0.000** | **0.000** | 0.083 |  |  |  |  |  |  |  |  |
| **Moral** | High | **0.000** | **0.000** | **0.000** | **0.000** | **0.000** | **0.000** | 1.000 | 0.742 | **0.000** | **0.000** |  |  |  |  |  |  |  |
|  | Low | **0.000** | **0.000** | **0.000** | **0.000** | **0.000** | **0.000** | 0.811 | 0.933 | **0.000** | **0.000** | 0.876 |  |  |  |  |  |  |
| **Rational** | High | **0.000** | **0.000** | **0.000** | **0.000** | **0.001** | **0.006** | **0.000** | **0.001** | **0.000** | **0.000** | **0.000** | **0.000** |  |  |  |  |  |
|  | Low | **0.000** | **0.000** | **0.000** | **0.000** | **0.000** | **0.000** | **0.001** | **0.007** | **0.000** | **0.000** | **0.001** | **0.003** | 0.349 |  |  |  |  |
| **Counter-intuitive** | High | **0.000** | **0.005** | **0.002** | **0.007** | **0.000**  P: 1.000 | **0.000**  P: 0.643 | **0.000**  M: 0.089 | **0.000**  M: 0.245 | **0.000** | **0.000** | **0.000**  M: 0.108 | **0.000**  M: 0.179 | **0.004**  M: 0.060 | **0.000**  M: 0.239 | **0.000** |  |  |
|  | Low | **0.000** | **0.000** | **0.000** | **0.000** | **0.000**  P: 0.402 | **0.000**  P: 0.720 | **0.000**  M: 0.235 | **0.000**  M: 0.504 | **0.000** | **0.000** | **0.000**  M: 0.272 | **0.000**  M: 0.402 | **0.008**  P: 0.108 | **0.008**  M: 0.087 | **0.001**  S: 0.549 | **0.000** |  |
| **Unbiased** | High | **0.000** | **0.000** | **0.000** | **0.000** | **0.004** | 0.053 | **0.000** | **0.000** | **0.000** | **0.000** | **0.000** | **0.000** | 0.086 | 0.002 | **0.001**  P: 0.061 | **0.000**  P: 0.501 |  |
|  | Low | **0.000** | **0.000** | **0.000** | **0.000** | **0.000** | **0.000** | **0.000** | **0.000** | **0.000** | **0.000** | **0.000** | **0.000** | 0.816 | 0.117 | **0.009** | **0.002**  P: 0.085 | **0.000** |

*p*-values are adjusted for multiple comparisons using the Benjamini and Hochberg method to control for false discovery rate. Bold values are significant at the *α* = 0.05 level. Each of the three counterintuitive types were tested separately, but average *p­-*values across the types are presented for clarity except for differences that represent changes in significance (represented as “B” for biology, “M” for mentality, “P” for physicality, or “S” for all comparisons of the same counterintuitive type across the two prestige conditions).

**Supplementary Table S5. Twenty best-supported models of proposition recall.**

| Name | Model | df | logLik | AIC | ∆AIC | w |
| --- | --- | --- | --- | --- | --- | --- |
| Significant variables from full model without income (“A”), with gender | present ~ firstStory + prestige + social + survival + emotionalNegative + counterintuitiveDomain + gender + memory | 14 | -26415.45 | 52858.91 | 0.00 | 0.116 |
| A with gender and line number | present ~ firstStory + line + prestige + social + survival + emotionalNegative + counterintuitiveDomain + gender + memory | 15 | -26414.46 | 52858.92 | 0.01 | 0.115 |
| A with gender and moral | present ~ firstStory + prestige + social + survival + emotionalNegative + moral + counterintuitiveDomain + gender + memory | 15 | -26414.61 | 52859.22 | 0.31 | 0.099 |
| A with gender and positive emotional | present ~ firstStory + prestige + social + survival + emotionalPositive + emotionalNegative + counterintuitiveDomain + gender + memory | 15 | -26414.68 | 52859.36 | 0.45 | 0.092 |
| A with gender and quadratic line number | present ~ firstStory + line + line^2 + prestige + social + survival + emotionalNegative + counterintuitiveDomain + gender + memory | 16 | -26414.08 | 52860.16 | 1.25 | 0.062 |
| Significant variables from full model without income (“A”) | present ~ firstStory + prestige + social + survival + emotionalNegative + counterintuitiveDomain + memory | 13 | -26417.34 | 52860.67 | 1.76 | 0.048 |
| A with line number | present ~ firstStory + line + prestige + social + survival + emotionalNegative + counterintuitiveDomain + memory | 14 | -26416.34 | 52860.68 | 1.77 | 0.048 |
| A with gender and country | present ~ firstStory + prestige + social + survival + emotionalNegative + counterintuitiveDomain + country + gender + memory | 15 | -26415.38 | 52860.76 | 1.85 | 0.046 |
| A with gender and town low prestige | present ~ firstStory + prestige + social + survival + emotionalNegative + counterintuitiveDomain + gender + townLowP + memory | 15 | -26415.41 | 52860.81 | 1.90 | 0.045 |
| A with gender and story | present ~ story + firstStory + prestige + social + survival + emotionalNegative + counterintuitiveDomain + gender + memory | 15 | -26415.44 | 52860.88 | 1.97 | 0.043 |
| A with gender and rational | present ~ firstStory + prestige + social + survival + emotionalNegative + rational + counterintuitiveDomain + gender + memory | 15 | -26415.45 | 52860.90 | 1.99 | 0.043 |
| A with moral | present ~ firstStory + prestige + social + survival + emotionalNegative + moral + counterintuitiveDomain + memory | 14 | -26416.49 | 52860.98 | 2.07 | 0.041 |
| A with positive emotional | present ~ firstStory + prestige + social + survival + emotionalPositive + emotionalNegative + counterintuitiveDomain + memory | 14 | -26416.56 | 52861.12 | 2.21 | 0.038 |
| A with quadratic line number | present ~ firstStory + line + line^2 + prestige + social + survival + emotionalNegative + counterintuitiveDomain + memory | 15 | -26415.96 | 52861.92 | 3.01 | 0.026 |
| A with gender and education | present ~ firstStory + prestige + social + survival + emotionalNegative + counterintuitiveDomain + gender + education + memory | 17 | -26413.99 | 52861.97 | 3.06 | 0.025 |
| A with town low prestige | present ~ firstStory + prestige + social + survival + emotionalNegative + counterintuitiveDomain + townLowP + memory | 14 | -26417.20 | 52862.41 | 3.50 | 0.020 |
| A with gender and ethnicity | present ~ firstStory + prestige + social + survival + emotionalNegative + counterintuitiveDomain + gender + ethnicity + memory | 16 | -26415.21 | 52862.41 | 3.50 | 0.020 |
| A with country | present ~ firstStory + prestige + social + survival + emotionalNegative + counterintuitiveDomain + country + memory | 14 | -26417.32 | 52862.65 | 3.74 | 0.018 |
| A with story | present ~ story + firstStory + prestige + social + survival + emotionalNegative + counterintuitiveDomain + memory | 14 | -26417.32 | 52862.65 | 3.74 | 0.018 |
| A with rational | present ~ firstStory + prestige + social + survival + emotionalNegative + rational + counterintuitiveDomain + memory | 14 | -26417.33 | 52862.67 | 3.76 | 0.018 |

Degrees of freedom (df), log likelihood values (logLik), and Akaike information criterion values (AIC), are provided for each model fit. ∆AIC is the change in AIC relative to the best-supported model. Akaike weights (w) were used in weighted model averaging and represent the relative likelihood of each model. The models displayed are all those with ∆AIC < 5 and w > 0.01. Random effects of participant and proposition number were included in all models.

**Supplementary Table S6. Full model-averaged coefficients for proposition recall.**

| Variable | Coefficient | SE | *p*-value | RVI |
| --- | --- | --- | --- | --- |
| intercept | -2.877 | 0.148 | **0.000** |  |
| story | 0.001 | 0.031 | 0.969 | 0.06 |
| firstStory | -0.699 | 0.045 | **< 0.001** | 1.00 |
| line |  |  |  |  |
| (linear) | -0.021 | 0.048 | 0.656 | 0.25 |
| (quadratic) | 0.005 | 0.027 | 0.843 | 0.09 |
| prestige | 0.152 | 0.023 | **< 0.001** | 1.00 |
| social |  |  |  | 1.00 |
| (none-basic) | 0.893 | 0.187 | **< 0.001** |  |
| (none-gossip) | 0.841 | 0.305 | **0.006** |  |
| survival | 0.620 | 0.216 | **0.004** | 1.00 |
| emotionalPositive | -0.055 | 0.186 | 0.768 | 0.13 |
| emotionalNegative | 1.129 | 0.254 | **< 0.001** | 1.00 |
| moral | -0.064 | 0.204 | 0.755 | 0.14 |
| rational | -0.001 | 0.056 | 0.985 | 0.06 |
| counterintuitiveDomain |  |  |  | 1.00 |
| (none-biology) | 2.023 | 0.336 | **< 0.001** |  |
| (none-mentality) | -0.280 | 0.274 | 0.307 |  |
| (none-physicality) | 0.610 | 0.359 | 0.089 |  |
| country (us-uk) | -0.003 | 0.045 | 0.938 | 0.06 |
| gender (female-male) | -0.234 | 0.207 | 0.259 | 0.71 |
| ethnicity |  |  |  | 0.03 |
| (white-poc) | -0.003 | 0.049 | 0.951 |  |
| (white-mixed) | 0.007 | 0.073 | 0.928 |  |
| townChildhoodSize |  |  |  | < 0.01 |
| (linear) | 0.000 | 0.008 | 0.995 |  |
| (quadratic) | 0.000 | 0.009 | 0.984 |  |
| (cubic) | 0.000 | 0.008 | 0.999 |  |
| townChildhoodLowP (false-true) | 0.006 | 0.068 | 0.928 | 0.06 |
| education |  |  |  | 0.03 |
| (linear) | 0.003 | 0.038 | 0.932 |  |
| (quadratic) | 0.006 | 0.044 | 0.899 |  |
| (cubic) | -0.007 | 0.046 | 0.885 |  |
| occupation |  |  |  | < 0.01 |
| (student-homemaker) | 0.000 | 0.017 | 0.998 |  |
| (student-production) | 0.000 | 0.019 | 0.997 |  |
| (student-trades) | -0.001 | 0.032 | 0.981 |  |
| (student-sales) | 0.000 | 0.015 | 0.996 |  |
| (student-service) | 0.000 | 0.016 | 0.991 |  |
| (student-professional) | 0.000 | 0.014 | 0.998 |  |
| income |  |  |  | < 0.01 |
| (linear) | 0.001 | 0.030 | 0.985 |  |
| (quadratic) | 0.003 | 0.076 | 0.969 |  |
| (cubic) | 0.000 | 0.026 | 0.989 |  |
| memory | 0.579 | 0.085 | **< 0.001** | 1.00 |

Relative variable importance (“RVI”) is the sum of Akaike weights (w) for all models that include that variable. Bolded *p*-values indicate statistically significant results at the 0.05 level.

**Supplementary Figures**


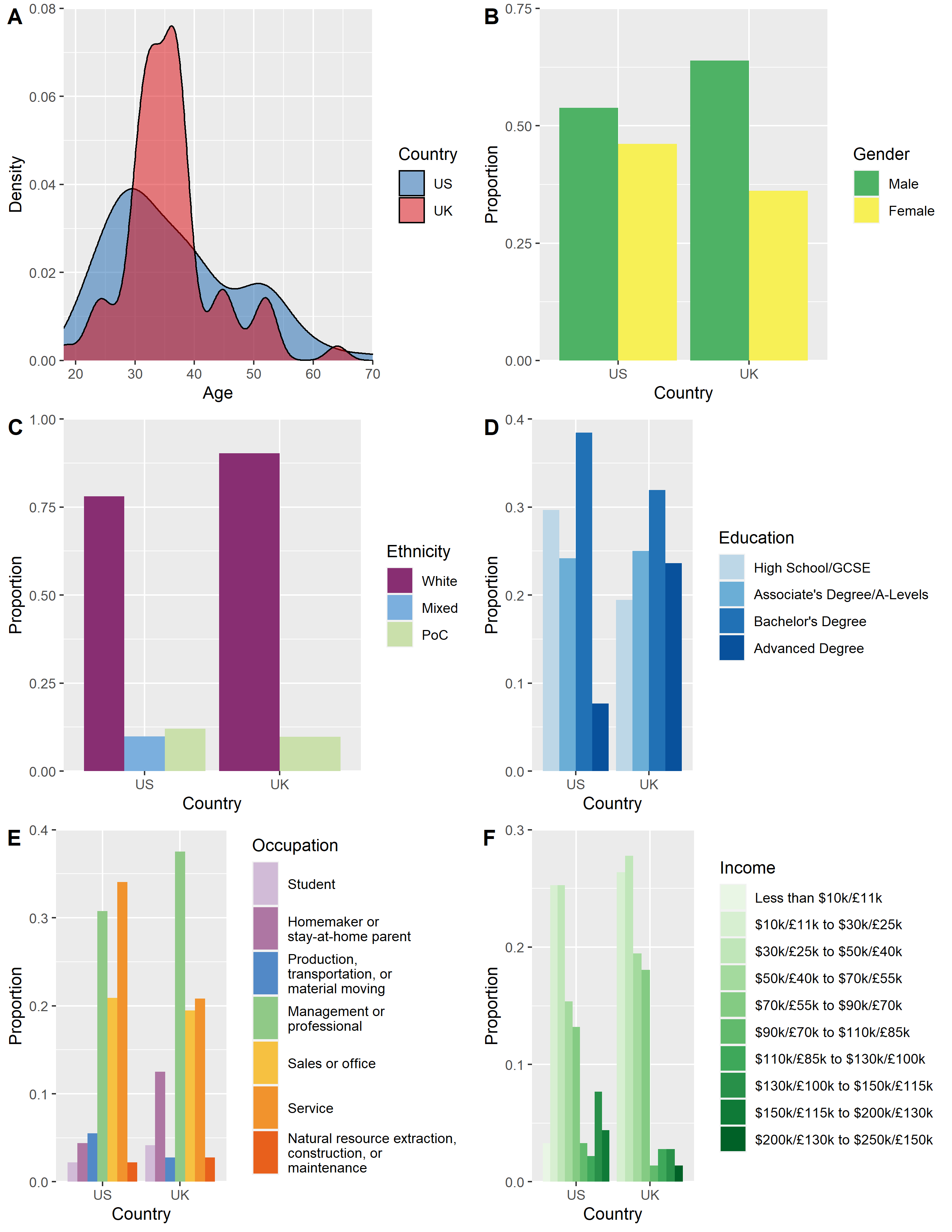


**Supplementary Fig. S1.** Sample demographics by country for: (A) age; (B) gender; (C) ethnicity/race; (D) education; (E) occupation; and (F) income. Some levels (such as the lowest income bracket) are not present in both country subsamples. Additional demographic variables not summarized here are provided in the data set.
